# Pulmonary artery injury during mediastinoscopy controlled without gauze packing

**DOI:** 10.1186/1749-8090-6-15

**Published:** 2011-02-08

**Authors:** Muneo Minowa, Masayuki Chida, Syunsuke Eba, Yuji Matsumura

**Affiliations:** 1 Ohta-Nishinouchi Hospital, Department of General Thoracic Surgery, Koriyama, Japan; 2 Dokkyo Medical University, Department of General Thoracic Surgery, Mibu, Japan; 3 Tohoku University Hospital, Department of Chest Surgery, Sendai, Japan

## Abstract

The most serious complication that can occur during mediastinoscopy is hemorrhage from large vessels in the mediastinum, whereas there are few articles relating to injury to major vessels. We describe a case of 77-year-old male with mediastinal lymphadenopathy, who underwent a mediastinoscopy procedure. When the pretracheal lymph nodes adjoining the right pulmonary artery were biopsied, a massive amount of bleeding spilled out through the scope. Immediately, the scope was removed from the body and the bleeding was controlled with digital compression at the skin incision. Then we closed the incision in a three-layer manner without any gauze packing in the mediastinum. Although some reports recommended gauze packing for massive bleeding during mediastinoscopy, we believe not all cases need gauze packing because bleeding from a low-pressure circulation system component into closed compartment, such as mediastinum, would cease without resulting in a large hematoma or pseudoaneurysm.

## Background

Although many studies have found that mediastinoscopy is extremely safe, the most serious complication that can occur is hemorrhage from the large vessels in the mediastinum. There are few articles relating to injury to major vessels during mediastinoscopy [1-5] and there are few articles relating to this condition in the provided Medline search. This is an underreported condition, since in any group of general thoracic surgeons, discussion do no more than gravitate to anecdotes relating to mediastinoscopy.

Some reports have recommended gauze packing for massive bleeding from large vessels [4,5]. In fact, there are times when compression with material like surgicel^® ^will be requires, and there might also be times when surgical exploration will be required. However, we speculated that bleeding from a low-pressure circulation system component, such as the superior vena cava or pulmonary artery, would cease without the use of such packing without resulting in a large hematoma or a pseudoaneurysm, because the mediastinum is a closed compartmental space separate from the pleural cavity or open air space. Herein, we report a case of injury to the right pulmonary artery during a mediastinoscopy procedure that was successfully controlled without gauze packing.

## Case Presentation

**Figure 1:**
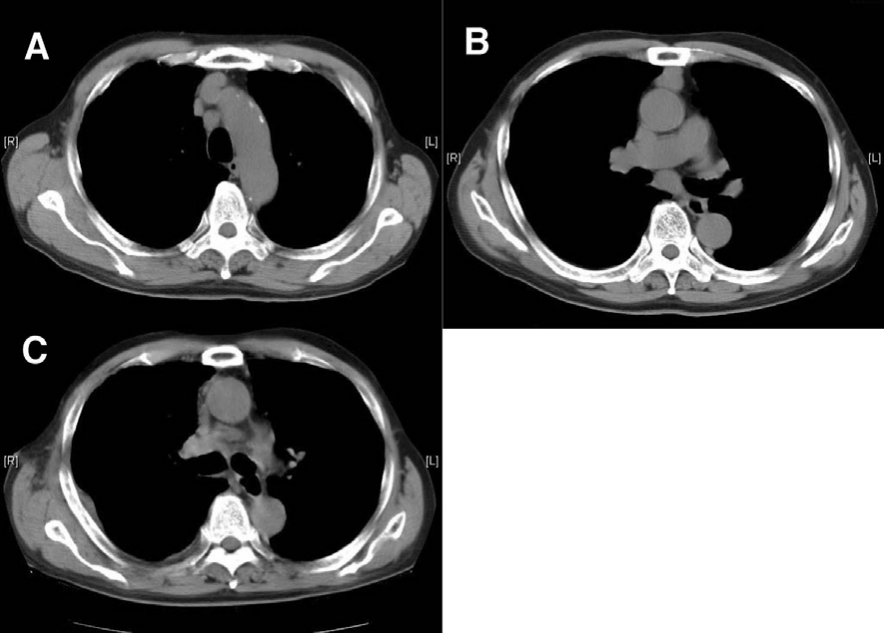
**CT scan images of (A) pretracheal lymph node, (B) subcarinal lymph node, and (C) mediastinum after hemorrhage from right pulmonary artery**. A small hematoma developed in the pretracheal space. Gauze packing was not done.

A 77-year-old male with mediastinal lymphadenopathy was referred to our hospital. Chest computed tomography (CT) scanning demonstrated #4R and #7 lymphadenopathy without tumors in the lung field (Figure 1). A gastrointestinal fiberscope examination found no malignancy in the upper gastrointestinal tract, while 18F-fluorodeoxy-glucose positron emission tomography showed a positive accumulation in the mediastinal lymph nodes without other suspicious lesions.

The patient underwent a video-assisted mediastinoscopy procedure in a supine position with a 5-cm cervical incision. The pretracheal lymph nodes adjoining the right pulmonary artery were found to be enlarged and carefully punch-biopsied several times. While a biopsy procedure was being performed, a massive amount of pulsate dark-red blood appeared and spilled out through the scope. Immediately, the mediastinoscope was removed from the body and bleeding from the incision was controlled with digital compression at the site of cervical incision adjacent to sternal notch closing the outlet of bleeding flow. Since the blood color was dark-red, we considered that the bleeding occurred from a low-pressure circulation system component, such as the pulmonary artery, and thought that it would cease in the mediastinum compartment after closure without making a large hematoma, even though the bleeding was massive in open air space. Digital compression was maintained for 10 minutes, and we confirmed that there were no changes of vital signs. Then we closed the incision in a three-layer manner without any gauze packing in the mediastinum.

**Figure 2:**
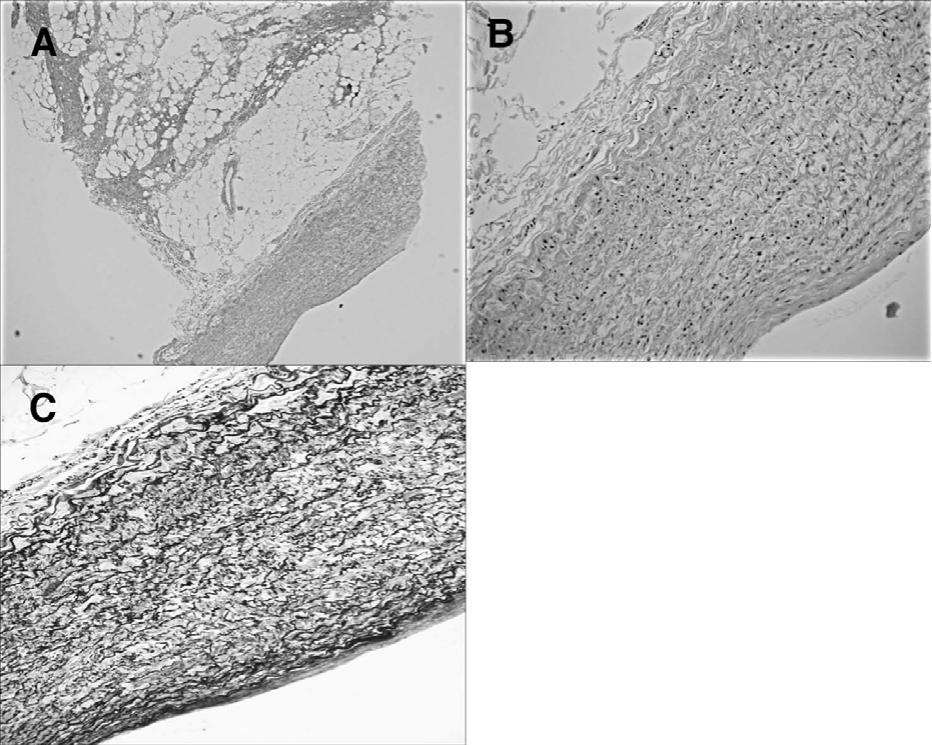
**Pathological findings of the biopsied specimen**. (A) Lymph node tissue and pulmonary artery wall in biopsied specimen (H&E, ×40). (B) Smooth muscle layer and endothelial layer (H&E ×200). (C) Smooth muscle layer of the artery (E&M ×200).

A pathological examination of the biopsied specimens revealed a whole arterial wall with a smooth muscle layer and vascular endothelium indicating that pulmonary artery wall was punched out (Figure 2). The bleeding came from the right main pulmonary artery, but not from the superior vena cava. A follow-up CT scan showed a small hematoma in the mediastinum (Figure 1C) that had not been observed before the mediastinoscopy procedure. Diagnostic specimens were not obtained during the mediastinoscopy, thus we performed a right thoracoscopic biopsy of a subcarinal lymph node two weeks later. There were no coagula seen in the subcarinal space. Squamous cell carcinoma metastasis in a subcarinal lymph node from an unknown origin was diagnosed and the patient underwent chemoradiotherapy.

## Discussion

The definition of major hemorrhage is unclear, though it generally includes bleeding greater than 500 mL, bleeding that requires a blood transfusion, or bleeding that requires exploration through a sternotomy or thoracotomy for control. Park et al. [4] defined major hemorrhage as any bleeding that requires an additional surgical incision for definitive control and reported several cases of bleeding from the pulmonary artery. In the present case, an injury to the pulmonary artery did not require additional exploration. However, it was not minor, as a whole arterial wall layer was punched out during a biopsy procedure.

Some reports have recommended gauze packing for massive bleeding from large vessels [4,5], including the superior vena cava and right pulmonary artery, each of which are considered to be low pressure circulating system components. If the site of bleeding adjoins the pleura, such as bleeding from the azygos vein or segmental pulmonary artery, bleeding may result in hemothorax and hemorrhagic shock. On the other hand, such bleeding will cease development into a hematoma when it occurs in the mediastinum and the bleeding site does not adjoin the pleura, because of the increased internal pressure.

When digital compression of cervical incision success to stop bleeding spilled from the outlet and then vital signs are stable, I think gauze packing may not be necessary. Unfortunately, when digital compression fails to control bleeding, gauze packing and/or surgical exploration will be required. Gauze packing following a cervical incision requires an additional operation to remove the gauze [5]. Absorbable material such as Surgicel^® ^may be useful for compression. The various approaches can be used for dealing with bleeding during mediastinoscopy. Sternotomy, right, or left thoracotomy can be chosen with or without cardiopulmonary bypass in relation to the side and nature of primary lesion.

Nagayasu et al. [6] reported that use of gauze packing for bleeding from the right pulmonary artery that occurred during a mediastinoscopy resulted in a 4-cm long laceration of the artery that required an additional thoracotomy and suggested that the tip of the mediastinoscope, which remained in the mediastinum for gauze packing, might gradually extend the length of the laceration. Gauze packing itself may have a risk. When a laceration of the pulmonary artery is as large as in that report, exploration is necessary to avoid pseudoaneurysm formation. However, a hole in the pulmonary artery will likely be small when a punch biopsy is the cause of bleeding. Therefore, we believe that gauze packing may not be necessary for bleeding from the right main pulmonary artery that occurs during mediastinoscopy when digital compression of a cervical incision definitely controls the bleeding and vital signs are stable.

## Conclusions

We experienced a case of injury to the right pulmonary artery during a mediastinoscopy procedure that was successfully controlled without gauze packing. In the majority of the cases, compression and patience can help stop bleeding.

## Consent

Written informed consent was obtained from the patient for publication of this case report and any accompanying images. A copy of the written consent is available for review by the Editor-in-Chief of this journal.

## Competing interests

The authors declare that they have no competing interests.

## Authors' contributions

MM was an operator of the surgery. MC supervised strategy for this accidental bleeding and drafted the manuscript. SE carried out data collection and helped to draft the manuscript. YM participated in coordination and helped to draft the manuscript. All authors read and approved the final manuscript.
